# What's Shared in Movement Kinematics: Investigating Co-representation of Actions Through Movement

**DOI:** 10.3389/fpsyg.2018.01578

**Published:** 2018-08-28

**Authors:** Matilde Rocca, Andrea Cavallo

**Affiliations:** ^1^Department of Psychology, University of Torino, Turin, Italy; ^2^C'MoN, Cognition, Motion and Neuroscience Unit, Fondazione Istituto Italiano di Tecnologia, Genova, Italy

**Keywords:** joint motor tasks, kinematics, co-representation, movement styles, social interaction

## Introduction

In recent years, psychological research has shown a growing interest in the study of human social interaction. This has led researchers to develop new paradigms and to formulate new theories about how people adjust minds and bodies when interacting with each other (Schilbach et al., 2013; Gallotti et al., 2017). One intriguing question that arises when dealing with social interactions concerns what information actors share about each other when involved in a joint action. One of the most influential theories in this field states that, given the fundamental social nature of joint actions, people have the tendency to represent and map both one's own and others' task demands (Sebanz et al., 2003, 2005). However, this view has recently been challenged by proponents of the “referential coding account” who have criticized the apparent nonsocial nature of the tasks and methodologies used to formulate and support the co-representation theory (Dolk et al., 2011, 2014).

In the present opinion article, we briefly describe the experimental paradigms often employed to study the co-representation theory (section Co-representation theory: proponents and opponents). Then, we illustrate potential methodological issues related to these paradigms (section A methodological problem), and finally we propose a new strategy, based on the characterization of movement kinematics, to address the open question about what is shared in shared actions (section A motor solution).

## Co-representation theory: proponents and opponents

Investigating joint performance requires researchers to focus on interactive experimental settings, trying to overcome the long-lasting trend of studying humans in lonely environments (De Jaegher et al., 2010; Schilbach et al., 2013). To this end, Sebanz et al. (Sebanz et al., 2003) proposed a social version of a well-known individual Stimulus-Response Compatibility (SRC) paradigm: the Simon Task.

In the *joint* version of the task, two stimulus-response mappings of a two-choice task are distributed between two agents (e.g., Agent 1 presses for green squares; Agent 2 presses for red squares). Even with no need of taking the other's mapping into account, the results highlight an interference effect between a task-irrelevant aspect of the stimulus (e.g., its position on the screen) and a task-relevant aspect of the response (e.g., the position of the button to press). The similarity with the original Simon effect led researchers to formulate the *co-representation theory*, which states that, given the social nature of joint actions, people tend to co-represent automatically each other's portion of the task in a functionally equivalent way (Sebanz et al., 2003, 2005). This theory has received support from many other studies that have used SRC tasks to test its assumptions (e.g., Atmaca et al., 2008, 2011; Elekes et al., 2016).

The co-representation theory has nevertheless received criticism. Some authors have argued that the behavior people display during the joint Simon task derives from a universal information-processing rule, having little to do with social skills (Dolk et al., 2011, 2014). Different studies have demonstrated that a nonsocial attention-attracting event, such as a Japanese waving cat, elicits the very same behavior observed in the joint Simon task (Dolk et al., 2013; Puffe et al., 2017). The main idea, expressed by opponents of the co-representation theory in what they call the *referential coding account*, is that the other person's action simply provides a spatial reference for one's own action, in the same way as any sufficiently salient event would do.

These two perspectives seem to be hardly reconcilable, lying on contrasting interpretations. The debate thus appears to have reached a stalemate, and the co-representation theory is facing an unexpected impasse.

## A methodological problem

It is worth noticing that the referential coding account does not intend to deny the social nature of joint performances: what the authors claim as nonsocial is the behavior that arises from joint SRC tasks used to investigate the co-representation theory (e.g., Dolk et al., 2011, 2014; Yamaguchi et al., 2018). The referential coding account is in fact a nonsocial way to explain the observed effects, which thus sometimes fall in an interpretational ambiguity.

This consideration raises a methodological problem. Two possible issues may in fact concern the use of SRC tasks in investigating co-representations: one is interpretational, one is practical. Both issues stem from the task that the two participants perform, which is for both a key press. This type of response is described as *discrete*, and is often contrasted with *continuous* responses (e.g., Song and Nakayama, 2009).

The interpretational issue relates to the poorness of the actions performed. Investigating joint performance with a task that involves discrete responses seems to reduce the social nature of the interaction. Using such a simple task is surely helpful in controlling the experimental setting, yet it pays the cost of dealing with an unnatural social setting. In daily environments, our social partners engage in actions that are much more complex, which we understand and predict (for a review see Springer et al., 2012; Hasson and Frith, 2016). Therefore, joint SRC tasks restrict the focus to a partner's action that may be too minimal to highlight a social effect.

The practical issue concerns the dependent measure obtained from joint SRC tasks: response time (RT). Although RT measures have helped to infer several aspects of human cognitive processes, it is well established that they restrict the investigation to a unidimensional assessment of behavior, without the opportunity of accessing the “continuity of the mind” (e.g., Spivey and Dale, 2004; Song and Nakayama, 2009). In joint SRC tasks, RTs show an interference effect, which suggest that we represent the other person's task and that this representation weakens our performance. However, RTs do not allow to access the content of this representation, limiting in a way the investigation of the phenomenon. For example, to coordinate with others, we must consider not only *what* movements others are doing, but also *how* they are moving (Keller et al., 2014; Gallotti et al., 2017). RTs can thus provide insightful information about the *what* component of co-representations, but they cannot be informative about the *how*–i.e., whether we also represent the specific movement styles of others' actions (but see Schmitz et al., 2017).

## A motor solution

To overcome the methodological issues that seem to affect joint SRC paradigms, here we propose a different experimental strategy that might shed light on the co-representation phenomenon.

We propose to turn to experimental paradigms that elicit a more complex and enriched overt motor activity. These joint *motor* tasks could help to address both the interpretational and the practical issues linked to joint SRC tasks.

On the interpretational level, dealing with a partner that makes complex movements can enhance the ecological validity of the experiments, bringing the setting closer to a real-life social interaction. Human movements present unique features that distinguish them from artificial-generated motions (Thompson and Parasuraman, 2012; Steel et al., 2014); furthermore, besides fundamental regularities (Viviani and Flash, 1995), individuals show specific movement styles (Ting et al., 2015; Koul et al., 2016). The exclusively human capability to understand, predict, anticipate, and adjust to how other people move establishes the profound social aspect of joint performances. We thus believe that, assuming the validity of the co-representation theory, the use of motor tasks could help to reject alternative nonsocial interpretations of joint SRC results.

On the practical level, movement kinematics might constitute a much more informative dependent measure than RTs, although caution must be taken when dealing with multivariate measures that provide huge amounts of data (e.g., high levels of false positives; Simmons et al., 2011). When investigating internal processes, some authors suggest to replace RT measures with dependent variables that are more fluid, continuous, and that can change over time (Freeman et al., 2011); movement kinematics could be a good candidate because of their capacity of reflecting the unfolding of internal dynamic processes over time (Song and Nakayama, 2009; Freeman et al., 2011). Indeed, despite the role played by inhibitory processes (for a review see Schall et al., 2017), human movements reveal a lot about both our external and our internal world. For example, movement kinematics have proven to be different depending on objects' size, shape, mass, and even texture and fragility (Weir et al., 1991; Castiello et al., 1992; Savelsbergh et al., 1996; Ansuini et al., 2015; for review see Jeannerod et al., 1995; Castiello, 2005). Even more interestingly, kinematic features encode information about more abstract internal states, including intentions (Cavallo et al., 2016; Becchio et al., 2018), decisions (McKinstry et al., 2008), numerical representations (Song and Nakayama, 2008), and other cognitive processes (Song and Nakayama, 2009; Freeman et al., 2011). Therefore, movement kinematics could be an adequate measure to investigate complex internal representations, like those of other persons' tasks and actions.

The characterization of human movement has already been extensively investigated in social interaction studies (Krishnan-Barman et al., 2017); however, these studies often focus on distinguishing between individual and social behavior, without fully addressing the question of whether and how we use information about the others to succeed in a joint action. A vast literature suggests that our movements are different in a social setting (Becchio et al., 2010; Krishnan-Barman et al., 2017), and that they are highly influenced by other people's movements (Blakemore and Frith, 2005; Heyes, 2011). This seems to indicate that other people's actions are actually represented in our brains when we act together; yet it remains unclear how specific these representations are, and how they come into play during joint performances: How and to what extent is the representation of others' task demands integrated within one's own motor system during joint actions? Does this representation include information about the others' motor behavior? Is this information specific to the confederate one is interacting with?

**Figure 1:**
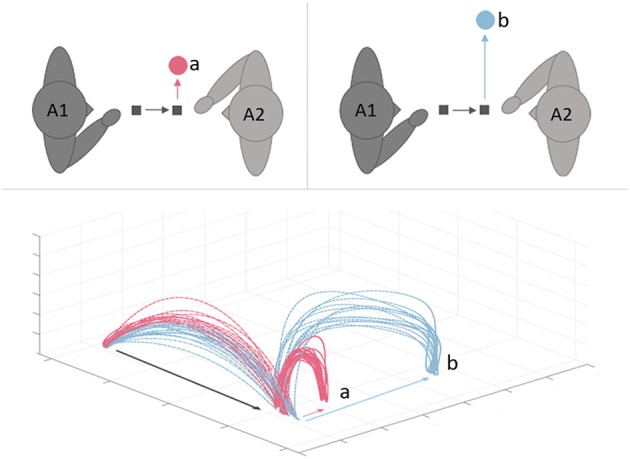
In the joint *motor* task pairs of participants are asked to perform sequential actions to reach a common goal. Agent 1 (A1) is asked to move an object from the starting position to an intermediate target area. Then, A2 grasps the object in the intermediate target area and places it in a final target area that varies across trials (e.g., different distance and size; upper panels). We expect that A2's task demands will be processed by A1. If so, kinematic profiles of A1 movements should encode information about the movement that A2 is *about to* make (lower panel).

To address these questions, we propose to use joint *motor* tasks involving participants in *sequential actions*, with the aim of reaching a common goal. A possible method could be to maintain the movement requirements of the first agent (A1) constant throughout the interaction, while manipulating those of the second agent (A2)–i.e., modifying the difficulty of A2's task, while keeping that of A1 constant. The kinematic profile of the first agent's movements could then be a good predictor of the movement that the second agent is *about to* make (Figure 1).

Compared to simultaneous actions, sequential motor tasks might increase the internal validity of the studies that aim to investigate co-representations, as they prevent from potential confounds caused by automatic imitation and motor contagion effects (Kilner et al., 2003; Heyes, 2011). Examining the similarity between the movement profiles of the two agents might in fact help on understanding how specific the representation of the other person's actions is, letting us begin accessing the content of co-representations.

Consider the kinematic modulation that occurs when an action is directed toward a small target: compared to large targets, movements toward small targets require greater precision, which is achieved through an earlier reach of the peak velocity and a longer deceleration phase (e.g., Marteniuk et al., 1987). We would in fact expect A2 to present an earlier time to peak velocity and a longer deceleration phase when his movement is directed toward a small, compared to a large target. If A1's velocity profile shows a modulation similar to that of A2, we would be facing two possible explanations. The first would suggest that A1 has formed a generic representation of A2's task: A2's targets might act as distractors for A1, producing an interference effect. The second would suggest that A1 has formed a detailed representation of A2's action, including kinematic information about the specific way in which A2 is going to move. In both cases, we would expect a positive correlation between the velocity profiles of the two agents. However, in the second case, the observed correlation would be higher than any other correlation obtained by permuting agents between pairs (e.g., correlation between A1 movements of pair *n* and A2 movements of pair *m*).

Another interesting aspect to explore would concern how the first agent's actions change over the course of the interaction. Building a representation of a person's actions may be a process that needs time and practice. The quantification of this kinematic adaptation could help to investigate how we learn to adjust to others in a joint task, and this would lead to explore the applicability of other theoretical models, such as associative learning (Catmur et al., 2009) and predictive coding (Kilner et al., 2007), to the joint action domain.

Furthermore, sequential motor tasks could provide a good tool to investigate whether co-representations arise exclusively in the joint action domain, where a common goal has to be achieved. Recent literature suggests that common goals might not be fundamental for creating social interactions (Gallotti et al., 2017). At the same time, other evidence points to consider common goals at the heart of reciprocal motor influence (della Gatta et al., 2017). In order to disentangle these different perspectives, it could be useful to investigate, through the manipulation of the instructions, whether and how others' motor representations change as a function of the presence/absence of a common goal.

## Conclusion

With the present opinion paper, we aimed at describing and facing the methodological issues connected to the paradigms currently used to support the co-representation theory. We presented an alternative approach to investigate the co-representation of actions, focused on the use of joint *motor* tasks.

We believe that shifting the attention to movement kinematics, and specifically to those emerging during sequential joint actions, could further the current understanding of how people successfully engage in joint performances. On the one hand, it is reasonable to think that the co-representation theory may gain from a motor approach the possibility of discarding the current criticism. On the other hand, a motor approach might provide the opportunity of bringing the investigation forward. Movement kinematics could in fact be a good tool to investigate not only how we *form* representations about others, but also how we *use* co-representations to coordinate and adjust to others.

## Author contributions

All authors listed have made a substantial, direct, and intellectual contribution to the work, and approved it for publication.

### Conflict of interest statement

The authors declare that the research was conducted in the absence of any commercial or financial relationships that could be construed as a potential conflict of interest.
